# A Comprehensive Review of Breast Fibroadenoma: Correlating Clinical and Pathological Findings

**DOI:** 10.7759/cureus.49948

**Published:** 2023-12-05

**Authors:** Sandeep Reddy Ramala, Suresh Chandak, Meenakshi S Chandak, Srinivasulareddy Annareddy

**Affiliations:** 1 General Surgery, Jawaharlal Nehru Medical College, Datta Meghe Institute of Higher Education and Research, Wardha, IND; 2 Surgery, Jawaharlal Nehru Medical College, Datta Meghe Institute of Higher Education and Research, Wardha, IND; 3 Respiratory Medicine, Jawaharlal Nehru Medical College, Datta Meghe Institute of Higher Education and Research, Wardha, IND

**Keywords:** prognosis, treatment options, diagnosis, histopathological characteristics, clinical presentation, breast fibroadenoma

## Abstract

Breast fibroadenomas, common benign conditions, exhibit distinct clinical and histopathological features. This review highlights clinical presentation and histology correlations, providing insights for healthcare providers. Palpable masses, pain, and changes in breast appearance align with glandular and stromal components, emphasizing accurate diagnosis. Mammography, ultrasound, and MRI guide tailored treatment decisions. Challenges in differentiating atypical fibroadenomas highlight the need for meticulous histopathological evaluation. Clinical implications stress patient-centered care, shared decision-making, and ongoing follow-up. Future research focuses on genetic investigations and long-term studies. A multidisciplinary approach to breast fibroadenomas ensures comprehensive care for improved outcomes in both medical and emotional aspects.

## Introduction and background

Breast fibroadenoma is one of the most common benign breast tumors frequently encountered in clinical practice. Characterized by a proliferation of epithelial and stromal components, fibroadenomas often present as palpable breast masses. These lesions predominantly affect women of reproductive age and are known for their clinical variability, with a wide range of presentations from asymptomatic findings to discomfort and anxiety [1-3].

The prevalence of breast fibroadenoma is highest in women of reproductive age. It is commonly found in women between the ages of 14 to 35, with a reported incidence of 27.6% in women aged 18-40 years. The incidence decreases with increasing age and is less common in post-menopausal women. In the adolescent population, the overall incidence of fibroadenoma is 2.2% [4]. Breast fibroadenomas were first described in the medical literature over a century ago, and their classification and understanding have evolved considerably since then. These tumors typically comprise both glandular (epithelial) and connective tissue (stromal) elements. Fibroadenomas' specific histological characteristics and cellular variants have been subjects of great interest to pathologists and clinicians alike [4].

Despite their benign nature, fibroadenomas have long been a topic of clinical importance due to their potential to mimic the clinical and radiological features of malignant breast tumors, leading to diagnostic challenges and decisions regarding management [3].

Accurately diagnosing and effectively managing breast fibroadenomas rely on seamlessly integrating clinical and pathological findings. Clinical assessment, including patient history, physical examination, and imaging studies, provides vital information for initial suspicion and evaluation [4]. However, with a complementary understanding of the histopathological features, a definitive diagnosis and appropriate therapeutic strategies may be attainable [4].

Correlation between clinical and pathological findings not only aids in accurate diagnosis but also informs treatment decisions. It enables healthcare providers to distinguish fibroadenomas from potentially more severe breast conditions, such as breast cancer. Additionally, understanding the variations within fibroadenomas helps tailor management approaches, as not all fibroadenomas are equal in their clinical behavior [5].

This comprehensive review aims to bridge the gap between clinical presentation and histopathological characteristics of breast fibroadenoma. By delving into the clinical manifestations and diagnostic methods used in identifying fibroadenomas, we aim to elucidate how these findings correlate with the histological features. This review will encompass an extensive analysis of the clinical aspects of fibroadenoma, ranging from its epidemiology and risk factors to the various diagnostic techniques employed in clinical practice. Concurrently, it will explore the intricate histopathological details of fibroadenomas, including their cellular composition, variants, and molecular markers. Importantly, we will examine the clinical implications of these histological nuances, shedding light on how they influence patient management and treatment decisions.

## Review

Clinical presentation

Definition and Classification of Breast Fibroadenoma

Breast fibroadenoma, often referred to as fibroadenoma, is a common benign breast tumor characterized by its dual composition of glandular (epithelial) and connective tissue (stromal) elements. These tumors are typically well-defined, mobile, and distinct from the surrounding breast tissue. Fibroadenomas can vary in size, texture, and presentation, and they are classified into several subtypes based on their histological characteristics, such as cellular, myxoid, or complex fibroadenomas [6].

Prevalence and Incidence Rates

Fibroadenomas are among the most prevalent benign breast tumors, considerably impacting public health. Fibroadenomas are common benign breast lesions usually present as a single breast mass in young women. Simple fibroadenomas have a reported incidence of 7-13% in women from adolescence through the mid-20s who present to specialty clinics. They are predominantly encountered in women, particularly those of reproductive age. Although they can develop at any stage of life, the highest incidence is typically observed between the ages of 15 and 35. The prevalence rates may vary across different populations and ethnic groups, with some studies suggesting a higher prevalence among African-American women [6].

Risk Factors Associated with Fibroadenoma Development

Hormonal factors: Hormonal dynamics, notably the influence of estrogen and progesterone, are pivotal contributors to the development of fibroadenomas. Hormonal fluctuations during critical life stages, including puberty, pregnancy, and the menstrual cycle, have been linked to an increased risk of fibroadenomas. The heightened hormonal activity during these periods may stimulate the growth of fibroadenomas within the breast tissue [2].

Family history: A family history of fibroadenomas or other benign breast conditions can be a significant risk factor. Individuals with close relatives who have experienced fibroadenomas may face an elevated risk of developing these lesions. This familial predisposition underscores the potential genetic underpinnings of fibroadenoma development [2].

Race and ethnicity: There appears to be some variability in the prevalence of fibroadenomas among different racial and ethnic groups. Studies have suggested variations in the incidence of fibroadenomas among individuals of different racial backgrounds. While this observation raises intriguing questions about genetic and environmental factors, further research is required to elucidate these disparities fully [2].

Body weight: Emerging research has explored potential associations between body weight or obesity and the risk of fibroadenoma development. Some studies have suggested a link between higher body weight and an increased risk of fibroadenomas. This association may be attributed to hormonal changes associated with obesity. However, this relationship's precise mechanisms necessitate additional investigation [2].

Clinical Symptoms and Signs

Pain: Fibroadenomas are commonly characterized by their painless nature. However, some individuals may encounter intermittent breast discomfort or tenderness, particularly in the premenstrual phase. This cyclical breast pain is attributed to hormonal fluctuations and is a notable clinical aspect associated with fibroadenomas [7].

Palpable masses: One of the hallmark clinical features of fibroadenomas is the presence of palpable breast masses. These masses typically exhibit distinctive characteristics. They are often smooth to the touch, well-circumscribed, and possess mobility within the breast tissue. Their size can vary considerably, ranging from a few millimeters to several centimeters, rendering them palpable during clinical examination [4].

Changes in breast appearance: Fibroadenomas may, in some instances, induce changes in the appearance of the breast. Such alterations might encompass breast asymmetry, distortion of breast contours, or visible changes in the breast skin. These changes are attributed to the size and location of the fibroadenoma within the breast tissue and are factors that healthcare providers consider during clinical evaluation [4].

Other Clinical Findings

While fibroadenomas predominantly exhibit benign characteristics, it is essential to acknowledge that their clinical presentation may occasionally mimic that of malignant breast tumors. As part of a thorough clinical assessment, healthcare providers diligently consider additional clinical findings. These may include skin retraction, nipple discharge, or axillary lymphadenopathy. These features warrant careful evaluation to rule out more concerning breast conditions, ensuring a comprehensive diagnostic approach [4].

Diagnostic methods

Clinical Examination

Assessment of breast masses: A paramount focus of clinical examination is the evaluation of breast masses. Healthcare providers carefully gauge any palpable breast masses' size, location, consistency, and mobility. These critical observations contribute to the characterization of the breast lesion, aiding in the diagnostic process. In the context of fibroadenomas, the clinical examination allows for identifying masses that align with the glandular and stromal components of these benign lesions. Fibroadenomas' smooth, well-defined, and mobile nature often becomes evident during palpation [8].

Changes in breast skin and nipple: Clinical examination extends beyond mass assessment to include a meticulous evaluation of changes in breast skin and nipple. The healthcare provider diligently notes any skin retraction, nipple discharge, or visible alterations in the breast skin. These findings may offer critical clinical clues that guide subsequent diagnostic steps. At the same time, these features may not be characteristic of fibroadenomas; their presence or absence aids in differentiating benign lesions from potentially malignant conditions [9].

Axillary lymph nodes: The clinical examination includes the assessment of axillary lymph nodes. Healthcare providers assess the axillary region for the presence of enlarged or palpable lymph nodes. Enlarged lymph nodes may indicate regional involvement, potentially raising concerns about malignancy [10].

Imaging Techniques

Mammography: Mammography is a standard imaging technique in breast cancer screening and diagnosis. While fibroadenomas are typically benign, they may appear as well-defined, round, or oval masses with smooth margins on mammograms. Calcifications within fibroadenomas are rare but can occasionally occur. Mammography benefits older women or those with dense breast tissue [11].

Ultrasound: Breast ultrasound is an essential imaging modality for characterizing breast lesions, particularly in younger women with dense breast tissue. Fibroadenomas typically appear as hypoechoic (dark) masses with well-defined margins on ultrasound. This imaging technique allows for real-time visualization and assessment of the vascularity within the lesion, aiding in diagnosis [12].

MRI: Breast MRI is used in specific cases, such as when there are diagnostic uncertainties or a need for better characterization of lesions. Fibroadenomas typically appear as enhancing lesions with well-defined margins on contrast-enhanced breast MRI. MRI is beneficial for evaluating the extent of disease and identifying multiple lesions within the breast [13].

Biopsy and Histopathological Assessment

Fine-needle aspiration (FNA): FNA involves using a thin, fine needle to aspirate a sample of cells or fluid from the breast mass. This minimally invasive procedure is commonly utilized to evaluate breast lumps and confirm the benign nature of lesions, including fibroadenomas. FNA is particularly valuable for assessing simple fibroadenomas. However, it may not always provide sufficient tissue for a definitive diagnosis, especially when dealing with complex or atypical fibroadenomas. Despite its limitations in some instances, FNA remains a helpful initial step in the diagnostic process, often guiding subsequent management decisions [14].

Core needle biopsy (CNB): CNB represents a more substantial and precise biopsy technique. It entails the removal of a larger tissue sample using a core needle, which is equipped with a cutting edge. CNB is frequently preferred for diagnosing fibroadenomas as it yields an adequate tissue specimen for detailed histological examination. This method allows for a more comprehensive evaluation of the lesion's characteristics, facilitating a precise diagnosis. CNB is particularly valuable when dealing with fibroadenomas presenting complexities or atypical features, as it is more likely to obtain a definitive diagnosis [15].

Excisional biopsy: In situations where fibroadenomas are exceptionally large, exhibit complex features, or raise concerns about malignancy, an excisional biopsy may be deemed necessary. This surgical procedure involves completely removing the entire fibroadenoma for histopathological evaluation. Excisional biopsy provides the most extensive tissue sample for analysis, ensuring a comprehensive assessment of the lesion's nature. While it is a more invasive approach than FNA and CNB, excisional biopsy is indispensable when a definitive diagnosis is crucial or surgical intervention is warranted [4].

Role of Molecular Markers and Genetic Testing

Genetic alterations, including MED12: Emerging research has identified specific genetic alterations, such as mutations in the MED12 gene, associated with some fibroadenomas. Understanding these genetic markers is a promising avenue for enhancing our comprehension of fibroadenomas at the molecular level. The MED12 gene mutations, in particular, have been observed in a subset of fibroadenomas, contributing to our knowledge of their genetic basis. These findings hold potential implications for diagnosis and may aid in distinguishing fibroadenomas from other breast conditions [16].

Diagnostic implications: Exploring genetic markers in fibroadenomas offers the prospect of improving diagnostic accuracy. Identifying specific genetic alterations associated with these lesions may provide additional tools for confirming their benign nature and distinguishing them from other breast conditions, including malignant tumors. This could enhance our ability to make precise diagnoses and guide appropriate clinical management [17].

Challenges and future research: Despite these promising developments, the routine use of genetic testing for fibroadenoma diagnosis still needs to be standard practice. Further research is needed to establish the clinical utility of genetic testing in the context of fibroadenomas. Challenges include the need for larger-scale studies, validation of genetic markers, and the integration of genetic testing into existing diagnostic protocols. As our understanding of the genetic underpinnings of fibroadenomas continues to evolve, ongoing research will be instrumental in determining the practical applications and benefits of genetic testing in clinical practice [18].

Pathological characteristics

Histological Features of Fibroadenoma

Epithelial and stromal components: A unique amalgamation of both glandular (epithelial) and connective tissue (stromal) components is at the core of fibroadenomas. The epithelial elements form acini or ductal structures, closely resembling the glandular architecture of the breast. In contrast, the stromal component comprises fibrous tissue as a structural scaffold. These two components intricately intertwine, granting fibroadenomas their characteristic histological appearance. Both glandular and stromal elements are a hallmark of fibroadenomas, defining their distinct composition [2].

Cellular Variants

Simple fibroadenoma: This variant represents the most common presentation. Simple fibroadenomas exhibit a relatively uniform appearance characterized by elongated, compressed glandular structures and minimal stromal cellularity. The glandular elements maintain their characteristic architecture within this variant [4].

Cellular fibroadenoma: Cellular fibroadenomas stand out due to their heightened stromal cellularity. The increased presence of stromal cells can resemble phyllodes tumors, another type of fibroepithelial breast tumor. This variant's elevated stromal cellularity is a distinguishing feature [19].

Myxoid fibroadenoma: Myxoid fibroadenomas are defined by their abundant myxoid stroma, imparting a gelatinous appearance to the lesion. The unique presence of myxoid material within the stromal component is characteristic of this variant [20].

Apocrine fibroadenoma: Apocrine fibroadenomas include apocrine cells, which are larger and display distinct cytoplasmic features compared to typical ductal epithelial cells. The presence of apocrine cells sets this variant apart and may influence its clinical behavior [21].

Immunohistochemistry Findings

Expression of estrogen and progesterone receptors (ER and PR): One of the prominent immunohistochemistry (IHC) findings in fibroadenomas is the expression of ER and PR in the lesion's epithelial and stromal components. This finding aligns with the hormone sensitivity of fibroadenomas. ER and PR indicate that these tumors respond to hormonal influences, particularly estrogen and progesterone. Hormonal fluctuations, such as those occurring during the menstrual cycle or pregnancy, can impact the size and behavior of fibroadenomas. The expression of ER and PR reinforces the understanding of fibroadenomas as hormone-responsive lesions [22].

Absence of HER2/neu (human epidermal growth factor receptor 2) overexpression: Another crucial IHC finding in fibroadenomas is the absence of HER2/neu overexpression. This characteristic distinguishes fibroadenomas from certain malignant breast tumors, particularly those where HER2/neu overexpression is a hallmark feature. The absence of HER2/neu overexpression provides reassurance that fibroadenomas are benign lesions and aids in their differentiation from more concerning breast malignancies [23].

Variable expression of specific markers like CD10: Immunohistochemistry may reveal variable expression of specific markers, such as CD10, in the stromal cells of fibroadenomas. CD10 expression can be a helpful distinguishing feature when differentiating fibroadenomas from other breast lesions. CD10 is an antigen found on the surface of specific cells, including stromal cells in fibroadenomas. Its variable expression pattern can aid pathologists in confirming the diagnosis of fibroadenomas and distinguishing them from other entities with distinct IHC profiles [24].

Atypical and Complex Fibroadenomas

Epithelial hyperplasia: Atypical or complex fibroadenomas may display epithelial hyperplasia, characterized by an increased glandular cellularity within the lesion. This hyperplasia can lead to a higher density of glandular structures, deviating from the typical fibroadenoma architecture. While epithelial hyperplasia raises concerns due to its potential association with malignancy, it remains essential to differentiate it from true breast carcinomas through precise histological evaluation [25].

Sclerosing adenosis: Complex fibroadenomas may feature sclerosing adenosis, marked by increased stromal fibrosis within the lesion. This fibrosis can create a denser, more intricate tissue structure, potentially posing diagnostic challenges. Recognizing and differentiating sclerosing adenosis within fibroadenomas is essential to ensure accurate classification and management [26].

Calcifications: Calcifications within atypical or complex fibroadenomas are another noteworthy feature. Calcium deposits may form within the lesion, adding to its complexity. While calcifications can raise concerns about malignancy, they must be carefully evaluated in the context of the lesion's overall histological characteristics and clinical presentation [27].

Cystic changes: Atypical or complex fibroadenomas may undergo cystic changes, forming cystic spaces within the lesion. These cystic spaces introduce an additional layer of complexity and may mimic cystic breast lesions. Accurate differentiation is crucial to determine the appropriate management approach [28].

Differential Diagnosis with Other Breast Lesions

Phyllodes tumors: Phyllodes tumors, though rare, share some features with fibroadenomas, leading to potential diagnostic challenges. These fibroepithelial tumors may exhibit overlapping clinical and imaging characteristics. However, histopathologically, phyllodes tumors tend to be more cellular and may display leaf-like growth patterns. The cellular nature and distinct growth patterns serve as key differentiators from fibroadenomas. Accurate diagnosis relies on histopathological examination and expertise in distinguishing these two entities [29].

Fibrocystic changes: Fibroadenomas can coexist with fibrocystic changes in the breast, posing diagnostic considerations. Fibrocystic changes encompass a spectrum of benign breast alterations, including cysts, fibrosis, and epithelial hyperplasia. It is essential to differentiate between fibroadenomas and these coexisting changes. While fibroadenomas are well-defined, solid masses with distinct margins, fibrocystic changes may present as cystic or fibrous areas within the breast tissue. Clinical and imaging correlation and histopathological assessment help distinguish between fibroadenomas and fibrocystic changes [30].

Breast cancer: In some cases, breast cancer lesions, particularly benign-appearing subtypes like tubular or lobular carcinomas, may resemble fibroadenomas. This similarity can pose diagnostic challenges, underscoring the importance of meticulous examination and clinical correlation. IHC plays a pivotal role in distinguishing between these entities. While fibroadenomas typically lack malignant markers, breast cancer lesions exhibit specific IHC profiles. Careful evaluation of clinical and imaging findings alongside IHC results is essential to differentiate benign fibroadenomas from potentially malignant breast cancer lesions, ensuring timely and appropriate management [31].

Correlation between clinical and pathological findings

How Clinical Presentation Aligns with Histopathological Features

Palpable masses: The hallmark clinical feature of fibroadenomas is the presence of palpable breast masses. These masses align closely with the histopathological composition of fibroadenomas, which consist of both glandular and stromal components. The smooth, well-defined, and mobile nature of these masses directly reflects the encapsulated nature of fibroadenomas. Histopathologically, the glandular component comprises epithelial cells, while the stromal component comprises connective tissue elements. The encapsulation of fibroadenomas within a fibrous capsule contributes to these lesions' distinct, palpable nature, distinguishing them from other breast conditions [2].

Pain: Although fibroadenomas are typically painless, some may experience breast discomfort or tenderness. This phenomenon aligns with the hormone sensitivity of fibroadenomas and the cyclical breast pain that may accompany hormonal fluctuations. Hormones, such as estrogen and progesterone, influence the size and behavior of fibroadenomas, leading to variations in pain perception. This clinical feature highlights fibroadenomas' dynamic nature and responsiveness to hormonal changes, underscoring the importance of considering hormonal influences in their clinical evaluation [32].

Changes in breast appearance: Fibroadenomas can induce changes in appearance, which often align with the size and location of the lesion within the breast tissue. These changes may manifest as breast asymmetry or distortion of breast contours. A fibroadenoma can alter the shape and appearance of the breast, potentially causing concern for patients. Understanding this correlation between clinical presentation and histopathological features helps healthcare providers reassure patients about these alterations' benign nature and potential reversibility with appropriate management [4].

Other clinical findings: In some cases, additional clinical findings such as skin retraction, nipple discharge, or axillary lymphadenopathy may raise concerns about malignancy. However, it's crucial to note that these features are more commonly associated with other breast conditions, particularly breast cancer. The absence of typical malignant features in fibroadenomas is confirmed through histopathological examination. The alignment of clinical presentation with histopathological features allows healthcare providers to differentiate fibroadenomas from more concerning breast conditions and make informed diagnostic decisions [33].

Impact of Imaging Techniques on Diagnostic Accuracy

Mammography: Mammography, a well-established imaging modality for breast assessment, holds significant importance in identifying fibroadenomas. It particularly shines in older women or those with dense breast tissue. The diagnostic accuracy of mammography is underscored by its ability to visualize fibroadenomas as distinct, well-defined masses. These masses typically exhibit round or oval shapes with smooth margins, a hallmark characteristic of fibroadenomas. Moreover, mammography can occasionally detect calcifications within fibroadenomas, a rare but noteworthy finding. The presence of calcifications aids in differentiation, contributing to enhanced diagnostic accuracy [34].

Ultrasound: Breast ultrasound is a highly effective tool for characterizing breast lesions, making it invaluable, especially in younger women with dense breast tissue. Its significant impact on diagnostic accuracy is attributed to several key features. Fibroadenomas typically appear as hypoechoic (dark) masses on ultrasound, displaying well-defined margins that facilitate their identification. This echo pattern is a strong indicator of fibroadenomas, bolstering diagnostic accuracy. Furthermore, the real-time imaging capability of ultrasound allows for dynamic assessment, enabling healthcare providers to observe vascularity within the lesion. The presence or absence of blood flow within the fibroadenoma is an additional diagnostic criterion, further enhancing accuracy [35].

MRI: Breast MRI, while reserved for specific cases, offers unparalleled visualization and characterization of breast lesions, including fibroadenomas. Its impact on diagnostic accuracy is particularly noteworthy due to distinctive features. Fibroadenomas typically manifest as enhancing lesions on breast MRI, characterized by well-defined margins. The ability of MRI to detect contrast enhancement serves as a valuable confirmation of the fibroadenoma diagnosis. Additionally, breast MRI excels in assessing the extent of disease, a critical aspect of diagnostic accuracy. It aids in identifying multiple lesions within the breast, contributing to a comprehensive understanding of the condition [36].

Diagnostic Challenges and Limitations

Atypical and complex fibroadenomas: One of the primary challenges in diagnosing breast fibroadenomas lies in the existence of atypical and complex variants. These variants may exhibit histopathological features that deviate from the typical characteristics of fibroadenomas and can resemble other breast lesions, including phyllode tumors or even breast cancer. Distinguishing between these entities requires meticulous histopathological examination and expertise. Accurate differentiation is crucial, as treating and managing atypical or complex fibroadenomas may differ significantly from typical fibroadenomas [37].

Cyclic changes: Fibroadenomas, being hormone-sensitive lesions, can exhibit cyclical changes in size and associated symptoms in response to hormonal fluctuations. This variability can sometimes create diagnostic challenges during clinical evaluation. Patients may report changes in the fibroadenoma's size or tenderness, which can mimic the characteristics of more concerning breast conditions. Healthcare providers must consider these hormonal fluctuations when evaluating fibroadenomas and rely on clinical, imaging, and histopathological data to diagnose accurately [38].

Imaging variability: While imaging techniques such as ultrasound and mammography are precious in diagnosing breast fibroadenomas, some may not display classic imaging features. This variability can make diagnosis less straightforward, as the imaging findings may need to align with the expected characteristics of a fibroadenoma. In such cases, clinical correlation becomes crucial, and a biopsy may be required to confirm the diagnosis definitively. It underscores the importance of a multidisciplinary approach where healthcare providers collaborate to ensure an accurate diagnosis, considering clinical and imaging findings [39].

Implications for Patient Management and Treatment Decisions

Diagnosis: Accurate diagnosis through histopathological assessment is fundamental in managing breast fibroadenomas. This process confirms the benign nature of fibroadenomas and rules out malignancy, providing patients with a definitive answer to their concerns. This diagnostic clarity alleviates patient anxiety and ensures the appropriate management approach is taken. Knowing that the lesion is benign can significantly reduce patients' emotional and psychological burden [38].

Treatment decisions: The combination of clinical evaluation, imaging techniques, and histopathological data is pivotal in guiding treatment decisions for breast fibroadenomas. When fibroadenomas are diagnosed, and their benign nature is confirmed, healthcare providers can make informed decisions regarding treatment options. Small, asymptomatic fibroadenomas with reassuring histopathological features are often managed conservatively with surveillance, offering patients the peace of mind that they do not require immediate interventions. In contrast, more significant or symptomatic fibroadenomas may warrant surgical excision to relieve discomfort or address cosmetic concerns. The alignment of clinical and histopathological information guides the selection of the most appropriate treatment strategy, ensuring that it is tailored to the individual patient's needs and clinical circumstances [40].

Benign nature: Healthcare providers are equipped to reassure patients regarding the benign nature of fibroadenomas. It is essential to emphasize that fibroadenomas are non-cancerous lesions and do not pose a threat to developing breast cancer. This reassurance alleviates patient anxiety and dispels concerns about malignancy, fostering a sense of security [38].

Potential for growth: Patients should be informed about the potential for fibroadenomas to grow over time. While fibroadenomas remain benign, they can increase in size. Notably, healthcare providers can convey that the risk of malignant transformation within fibroadenomas remains exceedingly low. This knowledge empowers patients to anticipate potential changes in their condition and understand that growth does not equate to malignancy [5].

Available treatment options: Education about available treatment options is integral to patient counseling. These options encompass a range of approaches, including watchful waiting and surveillance, surgical interventions, and minimally invasive treatments. By comprehending the nuances of each option, patients can actively engage in the decision-making process. Informed patients are better equipped to select a management approach that aligns with their preferences, clinical circumstances, and individual goals [41].

Shared decision-making: Patient-centered care emphasizes shared decision-making, wherein healthcare providers collaborate with patients to determine the most suitable course of action. This collaborative approach respects patients' autonomy, values, and preferences, allowing them to shape their healthcare journey actively. By engaging in shared decision-making, healthcare providers ensure treatment decisions are tailored to each patient's needs and priorities [42].

Treatment Options

Watchful Waiting and Surveillance

Observation and regular monitoring: For many small, asymptomatic fibroadenomas, immediate intervention may not be necessary. In such cases, watchful waiting and regular surveillance are often recommended. This approach involves periodic clinical breast exams and imaging studies, such as ultrasound or mammography, to monitor any changes in the fibroadenoma's size, characteristics, or behavior. By closely observing the fibroadenoma over time, healthcare providers can ensure that any potential developments are promptly detected and appropriately managed. This conservative approach prioritizes patient comfort and minimizes unnecessary interventions while maintaining vigilant oversight [43].

Patient education: Patient education is pivotal in the watchful waiting and surveillance approach. Patients are provided with comprehensive information about fibroadenomas, including their benign nature, potential for stability or regression, and minimal impact on overall breast health. Educating patients about the natural history of fibroadenomas empowers them to make informed decisions and understand the rationale behind the watchful waiting approach. Patients are also educated about the importance of promptly reporting any changes or new symptoms related to fibroadenoma, ensuring that any concerning developments are addressed without delay. This open communication between healthcare providers and patients fosters a partnership in managing the condition [44].

Surgical Interventions

Lumpectomy: A lumpectomy involves removing the fibroadenoma while preserving the surrounding breast tissue. This approach is typically chosen when the fibroadenoma is relatively large, causing discomfort or uncertainty about its nature. The primary goal of a lumpectomy is to alleviate symptoms, provide histopathological confirmation of the diagnosis, and ensure that the fibroadenoma is not harboring any malignant features. The excised tissue is sent for histopathological examination to confirm its benign nature. Lumpectomy offers the advantage of retaining most of the breast tissue and is associated with less scarring than more extensive surgical procedures [45].

Excisional biopsy: An excisional biopsy is essentially the complete removal of the fibroadenoma, akin to a lumpectomy. This approach is often chosen when there is a high degree of clinical or radiological suspicion surrounding the fibroadenoma or when complete removal is desired for peace of mind. An excisional biopsy serves diagnostic and therapeutic purposes, providing a definitive diagnosis while eliminating the fibroadenoma. The excised tissue is meticulously examined to confirm its benign nature and exclude malignant features. This procedure is particularly valuable in cases where patients and healthcare providers seek conclusive evidence of the fibroadenoma's nature [46].

Cryoablation: Cryoablation is a minimally invasive procedure that offers a conservative treatment option for selected cases of breast fibroadenomas. It involves freezing the fibroadenoma to destroy it while sparing the surrounding breast tissue. Cryoablation is typically suitable for cases where the fibroadenoma is small, accessible, and amenable to this minimally invasive approach. One of the critical advantages of cryoablation is its minimal invasiveness, resulting in reduced scarring and a shorter recovery period compared to traditional surgical interventions. While cryoablation offers an appealing alternative to surgery for certain patients, its suitability depends on the size and location of the fibroadenoma. It should be carefully evaluated on a case-by-case basis [47].

Emerging Minimally Invasive Treatments

Ultrasound-guided vacuum-assisted biopsy (VAB) stands out as a minimally invasive technique that leverages the precision of ultrasound guidance to target and remove fibroadenomas with great accuracy. This procedure involves using a vacuum-assisted device to obtain small tissue samples from the fibroadenoma, which are subsequently subjected to histopathological examination. Critical advantages of VAB include its minimal scarring, making it an appealing option for patients who prioritize cosmetic outcomes. Additionally, VAB is a compelling alternative to surgical excision, enabling diagnosis and treatment in a single procedure. Patients undergoing VAB typically experience a shorter recovery period and reduced post-procedure discomfort compared to traditional surgery [48].

Radiofrequency ablation (RFA) represents another innovative minimally invasive treatment for breast fibroadenomas. RFA involves using radiofrequency energy to heat and destroy fibroadenoma tissue, and it is particularly well-suited for treating small fibroadenomas. The procedure is guided by imaging, ensuring precise targeting of the fibroadenoma while preserving surrounding healthy breast tissue. Like VAB, RFA is known for its minimal invasiveness and minimal scarring. Patients who opt for RFA can expect a quicker recovery than surgical interventions [49].

These emerging minimally invasive treatments provide a significant advantage by offering patients with fibroadenomas alternatives to traditional surgery. The reduced invasiveness of these procedures results in less scarring, minimizes post-procedure discomfort, and allows for quicker recovery. However, it is essential to note that the suitability of these treatments may vary depending on factors such as the size and location of the fibroadenoma and individual patient preferences. Shared decision-making between healthcare providers and patients plays a pivotal role in determining the most appropriate treatment approach, ensuring that it aligns with the patient's clinical circumstances and personal goals [50].

Patient Counseling and Shared Decision-Making

Patient preferences: Understanding the patient's concerns, preferences, and goals is essential in selecting the most suitable treatment approach for breast fibroadenomas. Patients may have unique considerations, such as cosmetic concerns, fear of scarring, or personal values that influence their treatment decisions. A patient-centered approach ensures that the chosen treatment aligns with the patient's preferences and expectations [38].

Clinical evaluation: Accurate clinical assessment is the foundation of treatment decision-making. Healthcare providers must conduct a thorough clinical evaluation, including assessing the fibroadenoma's size, location, and associated symptoms. This evaluation guides treatment options and helps determine the appropriate course of action, whether it involves watchful waiting, minimally invasive procedures, or surgical intervention [38].

Risk-benefit assessment: Patients should be informed about each treatment option's potential benefits and risks. This includes a discussion of factors such as scarring, recovery time, and potential complications. A comprehensive understanding of the risks and benefits allows patients to make well-informed decisions that align with their priorities and values [51].

Long-term implications: It is crucial to educate patients about the long-term implications of their chosen breast fibroadenomas treatment. Patients should know the potential for recurrence and the importance of ongoing breast health management. Patients can actively participate in their health management and make decisions that consider both short-term and long-term outcomes by providing information about the expected course of their condition and the need for follow-up care [4].

Psychological impact: Breast lesions, including fibroadenomas, can significantly impact patients psychologically. Acknowledging the emotional and psychological aspects of breast health is essential. Healthcare providers should be attentive to patient anxiety and concerns, providing empathetic support and addressing fears or uncertainties. A patient-centered approach recognizes the importance of physical and emotional well-being in treating individuals with breast fibroadenomas [52].

Prognosis and follow-up

Natural History of Breast Fibroadenoma

Stability or regression: A prominent characteristic of fibroadenomas is their propensity to remain stable in size and clinical behavior over time. Many fibroadenomas do not exhibit significant changes in size or cause further symptoms or discomfort as they persist within the breast tissue. Some fibroadenomas even undergo spontaneous regression, particularly in younger individuals or in response to hormonal changes, such as those experienced during pregnancy or menopause. This natural regression can reduce the size and symptoms associated with fibroadenoma, ultimately improving the patient's quality of life [4].

Hormone sensitivity: Fibroadenomas are hormone-sensitive, which means they can respond to hormonal fluctuations by changing size and associated symptoms. This sensitivity is particularly evident during the menstrual cycle when hormonal variations are most pronounced. Fibroadenomas often enlarge and become more palpable or symptomatic during specific menstrual cycle phases. However, it is essential to note that these fluctuations do not indicate malignant transformation but rather a hormone-driven response. After menopause, when hormonal influences diminish, fibroadenomas typically stabilize or regress [47].

Low malignancy risk: The most reassuring aspect of fibroadenomas is their inherently low risk of developing breast cancer. Fibroadenomas themselves are benign lesions with a shallow risk of malignant transformation. However, it is essential to recognize that fibroadenomas can coexist with other breast conditions or lesions. Appropriate follow-up care, including clinical breast exams and imaging studies, is essential to monitor any concerning changes within the breast tissue. While fibroadenomas are benign, other concurrent conditions or lesions may require additional evaluation and management [4].

Risk of Recurrence

Spontaneous regression: One notable aspect of fibroadenomas is their potential for spontaneous regression. Some fibroadenomas may naturally shrink or resolve without any medical intervention. This phenomenon reduces the risk of recurrence, particularly in cases where the fibroadenoma regresses completely. Spontaneous regression can provide patients with relief from symptoms and decrease the likelihood of recurrence [53].

Age and hormonal factors: The risk of developing new fibroadenomas and the risk of recurrence can be influenced by age and hormonal factors. Younger individuals, especially those in the stages of puberty or pregnancy when hormonal fluctuations are more pronounced, may have a slightly higher risk of both new fibroadenomas developing and existing ones recurring. This hormonal influence is tied to the growth and development of fibroadenomas, which are hormone-sensitive lesions [54].

Incomplete excision: In cases where surgical excision is performed to remove a fibroadenoma, the risk of recurrence is generally very low when the entire fibroadenoma is successfully excised. Complete removal of the fibroadenoma eliminates the source of the lesion, reducing the likelihood of recurrence. However, incomplete excisions, where a portion of the fibroadenoma is left behind, may result in recurrence or the persistence of residual fibroadenoma tissue. Therefore, meticulous surgical techniques are essential to minimize the risk of recurrence in excised fibroadenomas [38].

Complex or atypical fibroadenomas: Complex or atypical fibroadenomas, which may exhibit variations from the typical histological characteristics, may have a slightly higher risk of recurrence than simple fibroadenomas. These variants can pose diagnostic and management challenges and may require closer follow-up to monitor for any changes or recurrence [55].

Long-Term Outcomes

Benign nature: Fibroadenomas maintain their benign nature throughout the patient's life. They possess an exceptionally low risk of undergoing malignant transformation, offering peace of mind to individuals diagnosed with these breast lesions. Patients can be reassured that the vast majority of fibroadenomas do not pose a threat of developing into breast cancer [6].

Stability: A noteworthy characteristic of fibroadenomas is their tendency to remain stable in size and clinical behavior over the long term. Many fibroadenomas neither increase in size nor cause further symptoms or discomfort as time progresses. This stability is particularly pronounced after menopause, as hormonal influences wane [6].

Improvement: In some instances, individuals may experience improved symptoms related to fibroadenomas. These benign breast tumors are known to undergo spontaneous regression, particularly in younger individuals or in response to hormonal fluctuations, such as during pregnancy or menopause. This natural regression can reduce the size and symptoms associated with fibroadenoma, further enhancing the overall quality of life for affected patients [6].

Minimal impact on breast health: Fibroadenomas have minimal impact on overall breast health. They do not increase the risk of developing breast cancer or other breast-related conditions. Individuals with fibroadenomas can expect their breast health to remain largely unaffected by these benign lesions [6].

Recommended Follow-up Protocols

Clinical breast exams: Regular clinical breast exams conducted by a healthcare provider are the cornerstone of adequate follow-up for fibroadenomas. These exams involve a thorough assessment of the breast, including the size, consistency, and characteristics of the fibroadenoma. Clinical breast exams are essential for tracking changes in the fibroadenoma's features and assessing its stability over time. They provide valuable clinical information that guides further management decisions [38].

Imaging studies: The choice of imaging studies depends on the patient's age and specific clinical context. Periodic mammograms or breast ultrasound may be recommended to assess the fibroadenoma and evaluate any potential changes. Mammography is particularly valuable for older individuals, while breast ultrasound is highly effective in assessing breast lesions, especially in women with dense breast tissue. These imaging studies enable the healthcare provider to visualize the fibroadenoma's characteristics and detect any new or unusual findings [56].

Patient education: Patient education is integral to follow-up care for breast fibroadenomas. Patients should be educated about breast self-exams and encouraged to perform them regularly. They need to understand the typical characteristics of their fibroadenoma, including its size, location, and associated symptoms. Patients should also be informed about the importance of promptly reporting any new symptoms or changes in the fibroadenoma to their healthcare provider. Patient education empowers individuals to participate in their breast health actively and facilitates early detection of any concerning developments [44].

Surgical follow-up: In cases where a surgical excision or biopsy has been performed to remove the fibroadenoma, post-operative follow-up appointments are essential. These appointments are necessary to ensure proper healing and assess for any signs of recurrence. Surgical follow-up may involve clinical breast exams and imaging studies to verify the complete removal of the fibroadenoma and confirm the absence of residual tissue. This meticulous approach aims to provide patients with peace of mind regarding the success of the surgical intervention [38].

High-risk populations: For individuals with a family history of breast cancer or other risk factors, more frequent follow-up and additional screening may be necessary. These high-risk populations require specialized attention and a personalized approach to follow-up care. Healthcare providers may recommend enhanced surveillance, genetic testing, or more frequent clinical breast exams and imaging studies to closely monitor the fibroadenoma and assess any potential changes in the breast tissue [57].

## Conclusions

In conclusion, our comprehensive review of breast fibroadenomas has shed light on the intricate interplay between clinical presentation and histopathological characteristics, offering valuable insights for healthcare providers and patients. We have emphasized the importance of accurate diagnosis, tailored treatment approaches, and patient-centered care. The correlations we have explored serve as a foundation for informed decision-making, reducing anxiety, and ensuring optimal outcomes. Looking ahead, further research avenues beckon, including genetic investigations and long-term studies. However, a multidisciplinary approach remains paramount in addressing breast fibroadenomas, encapsulating those affected's medical aspects and emotional and psychological well-being. Through this approach, we can provide reassurance, symptom relief, and a brighter outlook for individuals facing this common breast condition.
